# Pseudo-Gamma Spectroscopy Based on Plastic Scintillation Detectors Using Multitask Learning

**DOI:** 10.3390/s21030684

**Published:** 2021-01-20

**Authors:** Byoungil Jeon, Junha Kim, Eunjoong Lee, Myungkook Moon, Gyuseong Cho

**Affiliations:** 1 Applied Artificial Intelligence Laboratory, Korea Atomic Energy Research Institute, Daejeon 34507, Korea; bijeon@kaeri.re.kr; 2 Department of Nuclear and Quantum Engineering, Korea Advanced Institute of Science and Technology, Daejeon 34141, Korea; 3 Department of Environmental Radiation Monitoring and Assessment, Korea Institute of Nuclear Safety, Daejeon 34142, Korea; kimjh@kins.re.kr; 4 Decommissioning Technology Research Division, Korea Atomic Energy Research Institute, Daejeon 34507, Korea; leej0715@kaeri.re.kr; 5 Accelerator Development and Research Division, Korea Atomic Energy Research Institute-Korea Multi-Purpose Accelerator Complex, Gyeongju-si 38180, Korea

**Keywords:** plastic gamma spectrum, photopeak, full-energy peak unfolding, relative radioactivity prediction, deep learning, multitask model

## Abstract

Although plastic scintillation detectors possess poor spectroscopic characteristics, they are extensively used in various fields for radiation measurement. Several methods have been proposed to facilitate their application of plastic scintillation detectors for spectroscopic measurement. However, most of these detectors can only be used for identifying radioisotopes. In this study, we present a multitask model for pseudo-gamma spectroscopy based on a plastic scintillation detector. A deep- learning model is implemented using multitask learning and trained through supervised learning. Eight gamma-ray sources are used for dataset generation. Spectra are simulated using a Monte Carlo N-Particle code (MCNP 6.2) and measured using a polyvinyl toluene detector for dataset generation based on gamma-ray source information. The spectra of single and multiple gamma-ray sources are generated using the random sampling technique and employed as the training dataset for the proposed model. The hyperparameters of the model are tuned using the Bayesian optimization method with the generated dataset. To improve the performance of the deep learning model, a deep learning module with weighted multi-head self-attention is proposed and used in the pseudo-gamma spectroscopy model. The performance of this model is verified using the measured plastic gamma spectra. Furthermore, a performance indicator, namely the minimum required count for single isotopes, is defined using the mean absolute percentage error with a criterion of 1% as the metric to verify the pseudo-gamma spectroscopy performance. The obtained results confirm that the proposed model successfully unfolds the full-energy peaks and predicts the relative radioactivity, even in spectra with statistical uncertainties.

## 1. Introduction

Gamma spectroscopy is the quantitative study of the energy spectra of gamma-ray sources. Major applications of gamma spectroscopy include the identification and quantification of gamma-ray sources by analyzing their energy spectra. The full-energy peaks (FEPs), which correspond to the photon incident energy in the energy spectra, are typically used for the identification and quantification of gamma-ray sources.

Plastic scintillation detectors are low cost, easy to be manufactured in large volumes, and produce rapid measurements. They are also capable of neutron detection, which is one of the key points in radiation portal monitors for homeland security. This capability is very limited to the inorganic scintillators or silicon detectors. However, they exhibit poor spectroscopic characteristics, poor energy resolution, and lack FEPs in their spectra. Therefore, when analyzing the plastic gamma spectra, the Compton continuum is considered as the region-of-interest in pseudo-gamma spectroscopy, unlikely in general-gamma spectroscopy. Pseudo-gamma spectroscopy is defined as the identification and quantification of gamma-ray sources through the analysis of the Compton continuum in energy spectra.

Despite their disadvantages, plastic scintillation detectors are extensively used in radiation monitoring systems. Several studies have investigated the enhancement of the spectroscopic capabilities of plastic scintillators by employing signal processing [1,2,3,4,5,6,7,8,9] or pattern recognition techniques [10,11,12,13,14]. Most of these techniques can only identify the radioisotopes in the plastic gamma spectra, with the exception of two approaches. One approach involves the application of the inverse of the transfer matrix to unfold FEPs [8]. However, this method cannot be applied to spectra with poor counting statistics. The other approach, as presented in our previous study, involves the reconstruction of Compton edges using an autoencoder [14]. Unlike the former approach, this method can identify the energy of the incident gamma rays from the reconstructed Compton edges, even in spectra with poor counting statistics. However, this method does not permit the quantitative analysis of gamma ray sources.

In this study, we present a deep learning model that unfolds FEPs and predicts the relative radioactivity of the isotopes in the plastic gamma spectra using a multitask learning (MTL) scheme. Plastic gamma spectra are simulated using a Monte Carlo code and measured using a polyvinyl toluene (PVT) detector. The dataset is generated using a random sampling technique, similar to the procedure followed in our previous study. Furthermore, we implement a multitask model and perform hyperparameter tuning using Bayesian optimization. The performance of our model is verified for the measured spectra, and it is confirmed that the proposed model can unfold FEPs and predict the relative radioactivity of isotopes, even in spectra with statistical uncertainties, unlike in the inverse matrix approach.

## 2. Materials and Methods

### 2.1. Deep Learning Model

#### 2.1.1. Multitask Learning (MTL)

MTL is a learning method applied for building a machine learning or deep learning model to perform multiple tasks simultaneously. It has been used successfully in various machine learning applications, including language processing and computer vision [15,16,17,18,19]. A well-known advantage of MTL is the synergic effect among tasks, which is related to the performance of the deep learning model. Features that cannot be determined by training a model with a dataset for a single task can be determined by training a model with datasets for multiple tasks, thereby improving the performance of the model. This effect is known as the synergic effect among tasks, and these features are called synergic features.

To identify and quantify gamma-ray sources, it is sufficient to unfold the FEPs in the plastic gamma spectra because gamma spectroscopy is generally performed by analyzing the FEPs in the energy spectra. In addition to being a promising tool for the unfolding of FEPs, MTL is convenient and suitable for estimating the radioactivity of gamma-ray sources in gamma spectroscopy. Our model utilizes MTL for both tasks, achieving better performance than when using independent models for each task. The main advantages of MTL are its synergic effect, as discussed above, and overfitting robustness. Overfitting robustness is dependent on the number of tasks, and may not be realized in our case, wherein the number of tasks is only two. However, the synergic effect can be realized. Of the advantages of MTL, the synergic effect is more important than the overfitting robustness because synergy directly affects the model performance while there are several techniques to prevent the overfitting issues.

An autoencoder [20,21,22] is a generative model used in artificial neural networks, which generates an output signal whose dimension is identical to that of the input signal. It is comprised of two parts: An encoder and a decoder. In the encoder, the inputs are encoded into vectors of reduced dimensions called bottlenecks. In the decoder, the bottlenecks are decoded into the reconstructed signal. Autoencoders have been extensively used for dimension reduction in many applications [23,24]. Furthermore, they can be used for noise rejection. If noise signals are added to the training data used for training an autoencoder to reconstruct an input signal without noise, the autoencoder can be optimized to generate a function to reject noise signals.

**Figure 1:**
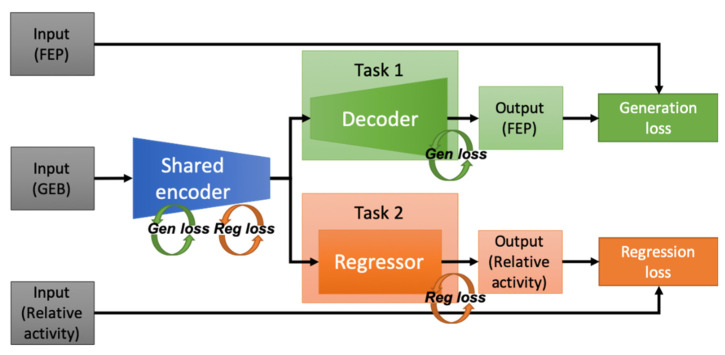
Configuration of the baseline model.

We employed the autoencoder structure as the skeleton of our baseline model to facilitate the generation of FEPs from plastic gamma spectra. In the encoder, the input plastic gamma spectrum is encoded in the bottleneck, which is connected to the decoder to generate the FEP spectrum. Another neural network is used for predicting the relative radioactivity of the gamma-ray source. The losses in training the MTL model are independently defined for each task: One is the generation loss in FEP unfolding, and the other is the regression loss in the relative radioactivity. As the encoder layers are shared for both tasks, the shared encoder is trained to minimize the generation and regression losses. The decoder layers and regression layers are trained to minimize the generation and regression losses, respectively. Figure 1 illustrates the overall configuration of the baseline model.

#### 2.1.2. Weighted Multi-Head Self-Attention

A deep learning model called the Transformer [25], first proposed for natural language processing, has been used in various machine learning applications [26,27,28,29]. The core modules in the Transformer model are multi-head attention and skip connection [30], denoted by ADD and NORM, respectively.

**Figure 2:**
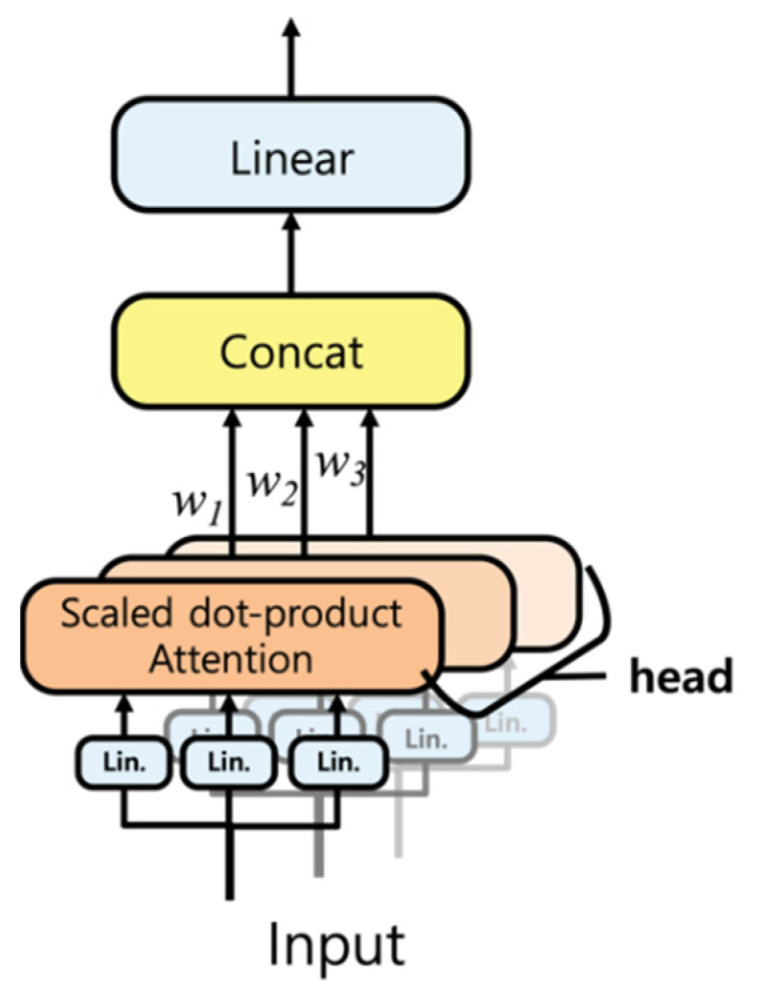
Weighted multi-head self-attention.

The attention mechanism attends to features that have the greatest influence on the results. In multi-head attention, multiple attention layers are arranged in parallel, and each layer is called an attention head. Multi-head attention ranks or assigns weights to the multiple features that influence the results. In multi-head self-attention, the results may not be meaningful, if the same inputs are provided to each attention head because the attention score is not trainable, but deterministically calculated. Such meaningless results are particularly noticeable when the input includes one-dimensional data, and this effect can be noticeable because less information is contained than multidimensional data. To prevent this issue, we propose weighted multi-head self-attention in which the output of each attention head is multiplied by head weights, which are trainable parameters in a neural network. With such multiplication, the attention scores from each attention head are selectively utilized, rendering the results of multi-head self-attention more meaningful. Figure 2 depicts a weighted multi-head self-attention module.

### 2.2. Dataset Generation

#### 2.2.1. Experimental Environment

As the plastic scintillation detector, a PVT crystal (EJ-220, EJ technology, Texas, United States) coupled with a photomultiplier tube (PMT; R2228, HAMAMATSU Photonics, Shizuoka, Japan), and socket assembly (E990-501, HAMAMATSU Photonics) were used. The used PVT crystal was cylindrical with a diameter of 30 mm and height of 50 mm. Optical grease was applied to the junction of the PVT and PMT, and the detector was wrapped in Teflon and black friction tape for optical shielding. For signal processing, an integrated pulse processor (DP5G, Amptek, Bedford, United Kingdom) that included a preamplifier, a shaping amplifier, and multichannel analyzer functions was used. Operating power was supplied by a high-voltage supplier (NHQ 224M, ISEG, Bautzen, Germany).

An aluminum dark box, with an internal width of 590 mm, height of 430 mm, and length of 890 mm, was used to reduce background gamma-ray radiation. The thickness of the aluminum layer was 5 mm. The detector was placed on the bottom plate of the box. As gamma ray sources, ^22^Na, ^54^Mn, ^57^Co, ^60^Co, ^109^Cd, ^133^Ba, ^137^Cs, and ^152^Eu (isotope products by Eckert and Ziegler) were used. Table 1 presents detailed information on the used gamma-ray sources. The sources were placed 1.25 cm away from the detector window and measured for various measurement periods. Energy calibration of the measured spectra was performed using the parametric optimization method [31].

#### 2.2.2. Monte Carlo Simulation

The general-purpose Monte Carlo N-Particle code MCNP 6.2 [33] was used for simulating plastic gamma spectra. The experimental environment, described above, was replicated in the simulation geometry, and the composition and densities of the materials were defined with reference to the material data report [34]. Pulse height tally along with a Gaussian energy-broadening (GEB) card was used to simulate the distribution of the energy deposition in the plastic scintillation detector. The GEB coefficients were calculated through parametric optimization [31] using the experimental spectra for a measurement period of one hour. Three cases of GEB coefficients were used: case 1: 0.0004 for “a”, 0.3704 for “b”, and −0.4999 for “c”; case 2: −0.0007 for “a”, 0.3944 for “b”, and −0.4999 for “c”; case 3: 0.0006 for “a”, 0.3548 for “b”, and −0.4999 for “c”. For these cases, the generated dataset was robust against slight variations in the shape of the plastic gamma spectra.

#### 2.2.3. Dataset Generation

A dataset for training, validation, and testing the developed model was generated by the random sampling technique using plastic gamma spectra, which are spectra with energy-broadening effects. To measure and simulate such spectra, eight gamma-ray sources were used. In the case of ^54^Mn, ^57^Co, and ^109^Cd, the spectra were measured for 24 h because of their low remaining radioactivity. For the other sources, the spectra were measured for one hour. All spectra were simulated with a history number of 10^9^. FEP spectra were generated based on the gamma-ray source information summarized in Table 1.

**Figure 3:**
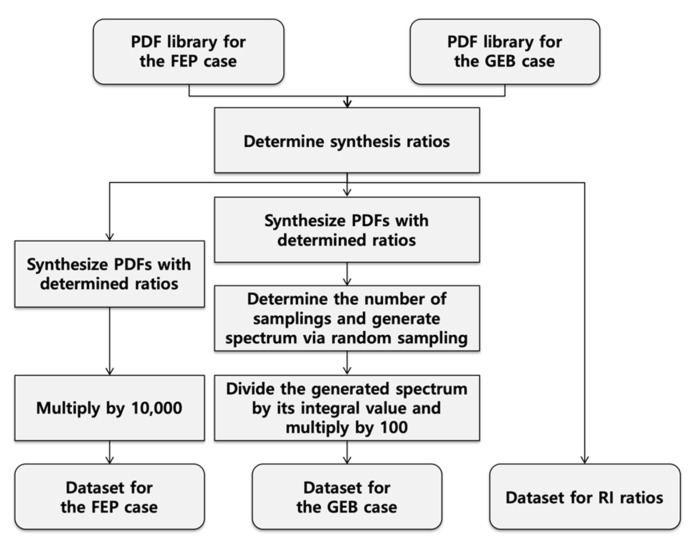
Dataset generation flowchart. By iterating the entire procedure 200,000 times, 200,000 spectra for the Gaussian energy-broadening (GEB) case, 200,000 spectra for the full-energy peaks (FEP) case, and 200,000 relative ratios were generated and used as a dataset.

The dataset was generated using the measured, simulated, and FEP spectra, and the synthesis ratios were randomly determined as described below. The measured, simulated, and FEP spectra were normalized by dividing by their integral values to produce normalized spectra that exhibit the characteristics of probabilistic density functions (PDFs). The normalized measured and simulated spectra were utilized for the libraries of the GEB cases, and the normalized FEP spectra were utilized for the libraries of the ideal cases. The dataset was generated as per the following procedure using the prepared PDF libraries. The dataset generation procedure is depicted in Figure 3 as a flowchart.

1. Determination of synthesis ratios

The PDF synthesis ratios were selected by dependent random number generation. Here, the dependent random number maintains the sum of the random ratios at unity to preserve the characteristics of the synthesized PDFs. For example, if the ratio for ^22^Na was determined as 0.22, the sum of the ratios of the others should be 0.78; if the ratio of ^57^Co was determined as 0.51, the others should exist in the range of 0–0.27. The number of radioactive sources and their synthesis ratios were determined using this dependent random number generation procedure. The synthesis ratios were stored in a dataset for relative ratios.

2. PDF synthesis with the determined ratios

The PDFs were determined using dependent random number generation to ensure that the synthesized PDFs retained the PDF characteristics, i.e., the sum of the synthesized PDFs was unity.

3. Random sampling for spectrum generation

The synthesized PDFs of the FEP spectra were used without changes, and those of the measured or simulated spectra were used for spectrum generation, performed in this step. The number of samplings to be performed was randomly selected in the 50,000–100,000 range using a uniform random number generator. Subsequently, a spectrum was generated through random sampling for the determined sampling number. As a result, a spectrum was generated with statistical uncertainties, and the total count of the spectrum was identical to the determined number of samplings.

4. Normalization

The spectrum generated in Step 3 was divided by its integral value and multiplied by 100. The generated GEB spectrum represents the interaction probabilities of each bin on a percentage scale. The FEP spectrum was multiplied by 10^4^ to represent the interaction probabilities on a scale of basis points, i.e., per ten thousand. The magnitudes of the multiplication factors for the GEB and FEP spectra were determined by trial and error. The generated GEB and FEP spectra were stored in a GEB and FEP case datasets, respectively.

By iterating the entire procedure 200,000 times, a paired dataset was generated, comprising 200,000 GEB spectra, 200,000 FEP spectra, and 200,000 relative ratios. Of the generated datasets, 160,000 were used as the training set, 30,000 were used as the validation set, and the remaining 10,000 were used as the test set.

### 2.3. Implementation of a Deep- Learning Model

#### 2.3.1. Baseline Model Implementation

An MTL model was implemented in the Python environment using the Tensorflow 2.0 library [35]. Mean averaged percentage error (MAPE) was used as the loss function for both tasks because it showed the best performance among the options considered (other loss functions considered included squared errors, logarithmic errors, categorical errors, and cross entropies) The Adam optimizer [36] was used to train the MTL model, and the number of epochs was set to 300. Furthermore, a check point function, which saves the best model during epochs by monitoring the validation loss, was implemented to prevent overfitting. Batch normalization layers [37] were added after each hidden layer to avoid the gradient-vanishing effect. For the baseline model, shown in Figure 1, hyperparameter tuning was performed using an open-source code for Bayesian optimization [38]. Table 2 presents a summary of the hyperparameter tuning results.

**Figure 4:**
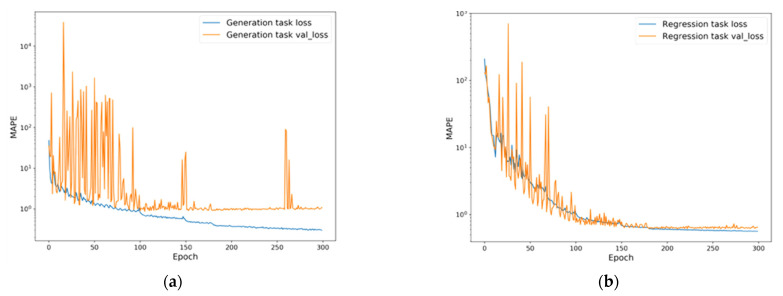
Training curves of the baseline model. Each figure represents training curve of (**a**) generation task; (**b**) regression task.

The baseline model, with the optimized hyperparameters, was trained using the generated training and validation sets during 300 epochs. As the shared encoder, decoder, and regressor were trained with the total, generation, and regression losses, respectively, we implemented a code-level training procedure. After activating the model check point function, the model with the minimum validation loss was saved and used as the final model. Figure 4 shows the training curve of the baseline model. Although MTL is known to be robust against the overfitting issues, overfitting occurred as shown in the figure. This was expected because the model was designed for only two tasks, and MTL overfitting robustness improves with more tasks. Therefore, we confirmed that it was necessary to use the model check point function. The trained model was evaluated using the test set. The evaluation results were 310.687% for the generation task and 34.225% for the regression task.

#### 2.3.2. Model Enhancement

**Figure 5:**
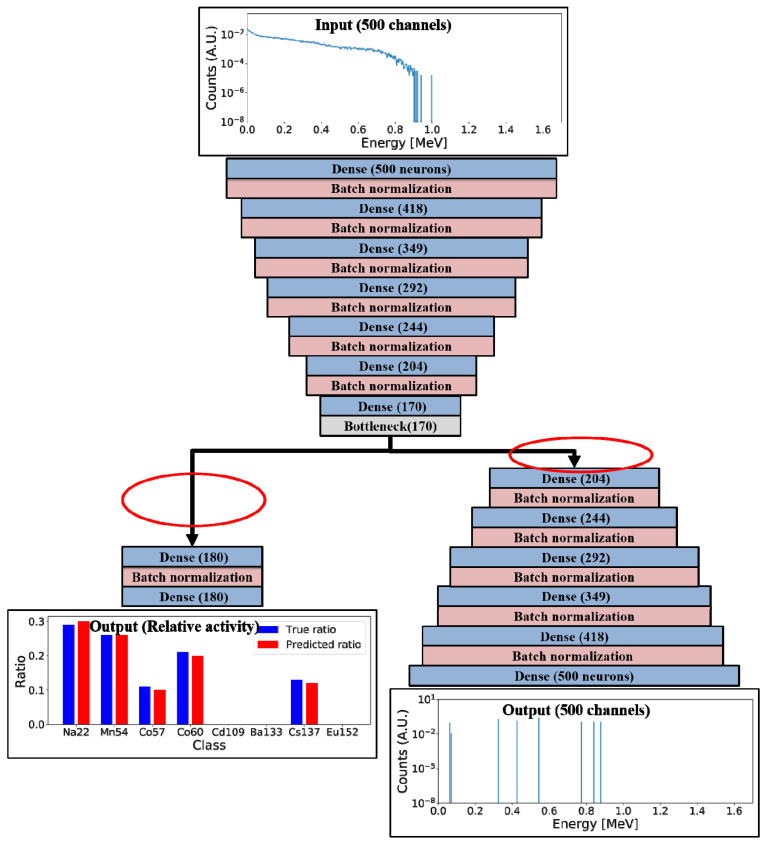
Structure of the baseline model for model enhancement (The red circles indicate the locations at which the deep learning modules are attached).

To improve the performance of the MTL model, we considered the application of deep learning modules such as the convolutional neural network (CNN), attention, residual connection, multi-head self-attention, and weighted multi-head self-attention. All these modules, except the CNN, can be applied to a model whose hidden layer dimensions are identical to one another. Therefore, the deep learning modules were considered attached after the bottleneck, i.e., at the beginning of each task-specific layer. Figure 5 depicts the structure of our model for model enhancement, and the locations at which the deep learning modules are attached.

## 3. Results

### 3.1. Model Enhancement

**Figure 6:**
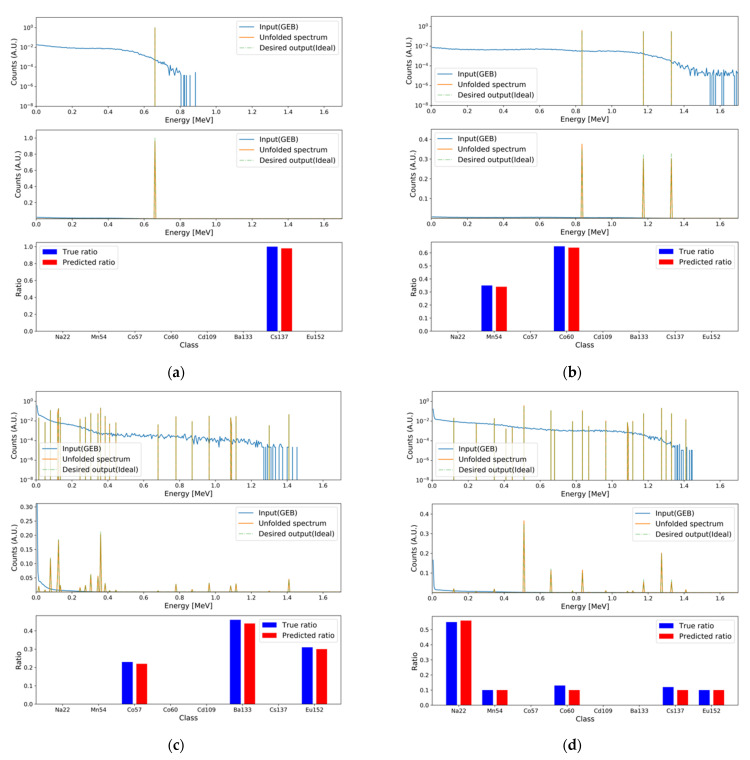
Examples of the generation and regression results for the test set. In each subfigure, the top and middle figures are plotted with log and linear scales for the *y*-axis because of the difference in scales for the FEP and GEB spectra. The mean averaged percentage errors (MAPEs) for the generation and regression tasks, respectively, were (**a**) 9.083 × 10^−2^ and 5.208 × 10^−1^; (**b**) 2.963 × 10^−2^ and 0.557; (**c**) 3.974 × 10^−1^ and 3.735; (**d**) 2.331 × 10^−1^ and 6.890.

**Figure 7:**
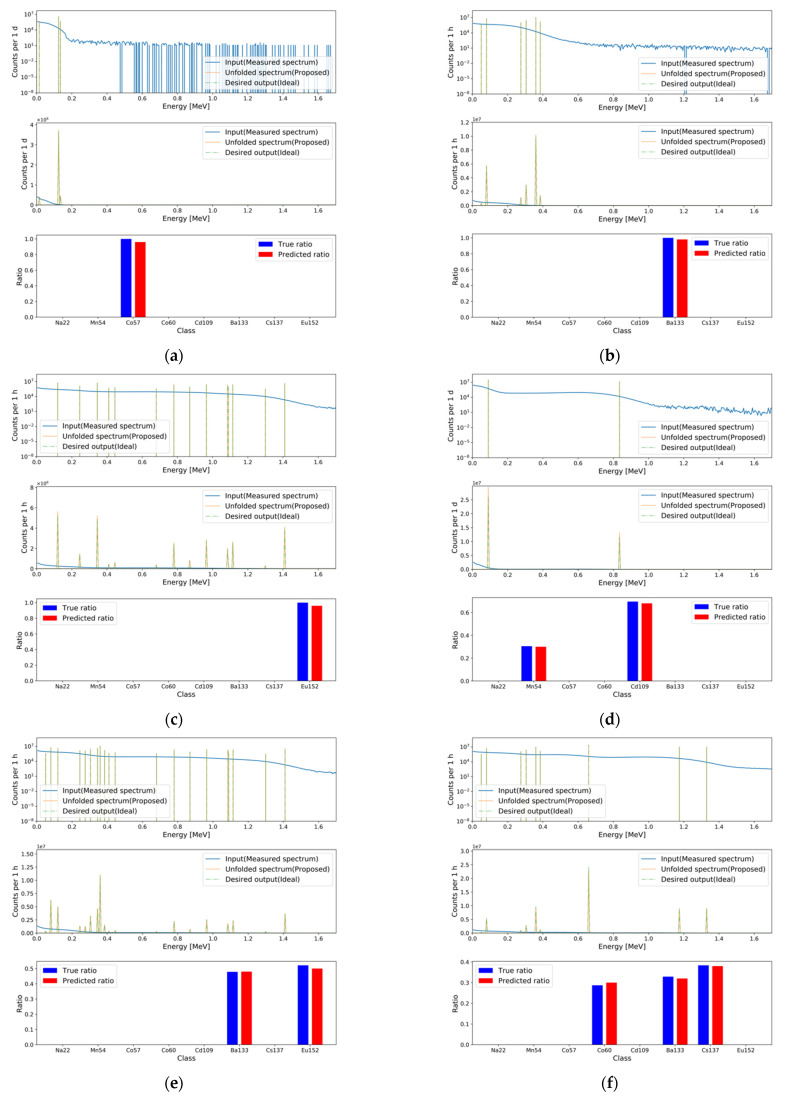
Examples of the generation and regression results for the measured spectra. The MAPEs for the generation and regression tasks, respectively, were (**a**) 1.539 × 10^−2^ and 5.001 × 10^−1^; (**b**) 3.616 × 10^−2^ and 2.499 × 10^−1^; (**c**) 1.483 × 10^−1^ and 5.001 × 10^−1^; (**d**) 5.531 × 10^−2^ and 4.637 × 10^−1^; (**e**) 5.752 × 10^−2^ and 5.508 × 10^−1^; (**f**) 6.046 × 10^−2^ and 1.029.

To validate the performance of the baseline model with deep learning modules, the following models were implemented: Deep neural network (DNN; baseline), DNN with attention, DNN with attention and skip connection, DNN with multi-head self-attention and skip connection, and DNN with weighted multi-head self-attention and skip connection (proposed). For the multi-head self-attention, the number of heads was set to five, and the dimension of the sublinear layers was set to 34. Furthermore, the following CNN models were implemented: CNN, CNN with attention, CNN with attention and skip connection, CNN with multi-head self-attention with skip connection, and CNN with weighted multi-head self-attention and skip connection (proposed). The length of the convolution filter was set to 25, and the number of convolution filters was set to 500. Global max pool layers were attached to each even-numbered hidden layer in the encoder with a pool size of two. Up-sampling layers were attached to each even-numbered hidden layer in the decoder with an up-sample size of two. The training setup for the above models was identical to that of the baseline model. To confirm the synergic effect among tasks, independent models for both tasks were implemented with identical hyperparameters as follows: DNN (two models) and DNN with the proposed module and skip connection (two models). The evaluation results using the test set for the implemented deep learning models are summarized in Table 3. DNN with the proposed module has the minimum generation loss among the implemented models. In the case of regression loss, the CNN model has the minimum value, but the generation loss of the CNN model is higher than that of the other DNN models. Therefore, DNN with the proposed module was selected as the final MTL model, even though it had the second lowest regression loss. In addition, the synergic effect was verified. As shown in Table 4, independent models of the DNN and DNN with the proposed module have higher losses in the generation and regression tasks compared to the MLT models of the DNN and DNN with the proposed module. Figure 6 and Figure 7 depict examples of the generation and regression results for the generated spectra in the test set, and for the measured spectra using the final MTL model, respectively.

### 3.2. Minimum Required Counts

One of the major performance parameters of radiation measurement systems is the minimum counts or radioactivity required to produce a sufficiently accurate result [13,39,40]. In this study, we defined the minimum required count (MRC) required by our MTL model to perform each task correctly. For the generation task, the MRC was defined as the minimum counts required by the proposed model to generate the correct FEPs from the measured spectrum of a single gamma-ray source. For the regression task, the MRC was defined as the minimum counts required by the proposed model to correctly predict the relative activity of a single gamma-ray source. To evaluate the MRC of each task, the evaluation set was generated as follows.

1. PDF calculation for the evaluation set

The measured spectrum of each gamma-ray source was normalized by dividing it by its integral value, and defined as the PDF for the evaluation set. For ^54^Mn, ^57^Co, and ^109^Cd, spectra with a measurement period of 24 h were used to calculate the PDFs because of their low remaining radioactivity. For the other sources, spectra with a measurement period of 1 h were used to calculate the PDFs.

2. Definition of the generation reference

For the measured spectra used in the PDF calculation, FEP generation was performed using the MTL model, and the results were defined as the generation reference for each gamma-ray source. The relative activity, set to unity for each gamma-ray source, was used as the regression reference.

3. Evaluation set generation

The spectra for the evaluation set were generated by random sampling using the PDFs calculated from the measured spectra. The number of samplings was in the range of 100–200,000 with an interval of 10. For each sampling set, 100 spectra were generated and used as the evaluation set.

**Figure 8:**
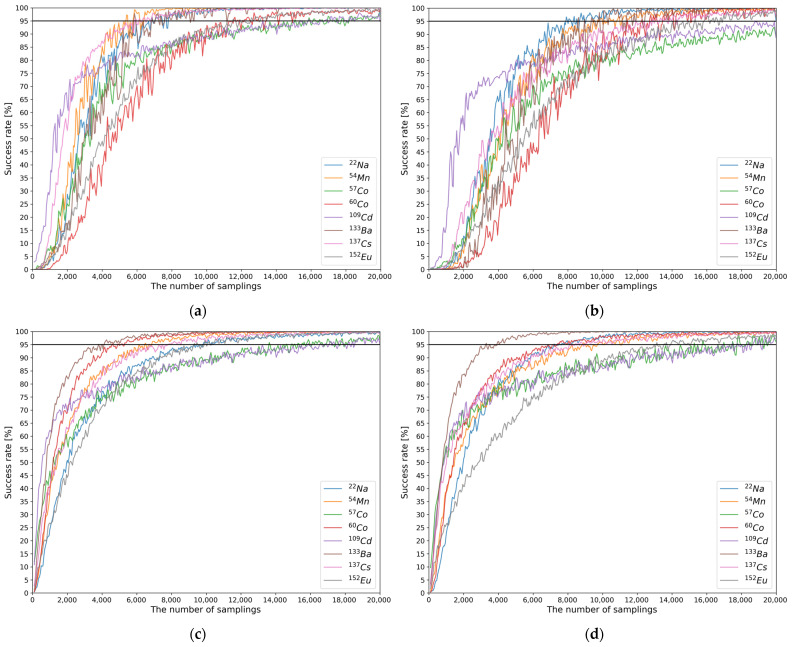
Success rates with the evaluation sets for the generation and regression tasks performed by the baseline model and our final MTL model. (**a**,**b**) Plots of the success rates in the generation and regression tasks, respectively, for the baseline model; and (**c**,**d**) plots of the success rates in the generation and regression tasks, respectively, for our final MTL model.

For the generated evaluation set, FEP generation and relative activity regression were performed using the developed MTL models. The MRC of each model was defined by evaluating the success rates for each sampling number as follows. The correct generation and regression results among 100 spectra were counted for each sample number, and the metric of correctness was defined using the MAPE with a criterion of 1%. That is, spectra whose MAPEs for the generation and regression results were less than 1% were considered successful. After the success rates for all the sampling numbers were calculated, the minimum sampling number with a success rate greater than 95% was considered as the MRC. For both tasks, the MRCs were evaluated for each gamma-ray source using the above procedure. To confirm that the proposed deep learning module, called weighted multi-head self-attention, contributed to performance improvement, we evaluated the MRCs for both the baseline model and our final MTL model. Figure 8 shows the success rates with the evaluation sets for both models in both tasks. The evaluated MRCs are summarized in Table 4.

As shown in Table 4, the MRCs of our final MTL model are generally lower than the baseline MTL model for both tasks. The MRCs differ slightly according to the task. For the baseline MTL model, the MRCs for the generation task were in the following order: ^54^Mn < ^22^Na < ^133^Ba < ^137^Cs < ^60^Co < ^152^Eu < ^57^Co < ^109^Cd. For the final MTL model, the MRCs for the generation task were in the following order: ^133^Ba < ^60^Co < ^54^Mn < ^137^Cs < ^152^Eu < ^22^Na < ^57^Co < ^109^Cd. The MRCs of the final MTL model were lower than that of the baseline MTL model for most of the gamma-ray sources, except for ^22^Na and ^54^Mn. In the case of the regression task, the MRCs of the baseline MTL model were in the following order: ^22^Na < ^54^Mn < ^133^Ba < ^137^Cs < ^60^Co < ^152^Eu < ^57^Co < ^109^Cd. The MRCs of the final MTL model were in the following order: ^133^Ba < ^60^Co < ^22^Na < ^137^Cs < ^54^Mn < ^152^Eu < ^109^Cd < ^57^Co. The MRCs of the final MTL model were lower than that of the baseline MTL model for most of the gamma-ray sources. In the case of ^54^Mn, the MRCs of the baseline and the final MTL models were identical. In both tasks, the MRCs for ^57^Co and ^109^Cd were relatively high. This may be because these gamma-ray sources emit low energy (below 100 keV) gamma rays. Because of the poor energy resolution of plastic gamma spectra, these low energy gamma rays are difficult to distinguish.

The gamma-ray sources with low MRCs differed slightly based on the task and model. Although each of these gamma-ray sources emits its own gamma-ray energy and their spectra can be clearly distinguished from the others, differences in the MRCs may arise due to the feature representations in the deep learning model.

To assess the MRC evaluation results, the background spectrum and the spectrum of each gamma-ray source were measured for 1–330 s corresponding to each MRC. FEP generation and relative activity regression were then performed by our MTL model. The measurement periods were adjusted such that the total net count of each background-subtracted spectrum was similar to each MRC within the statistical uncertainties.

**Figure 9:**
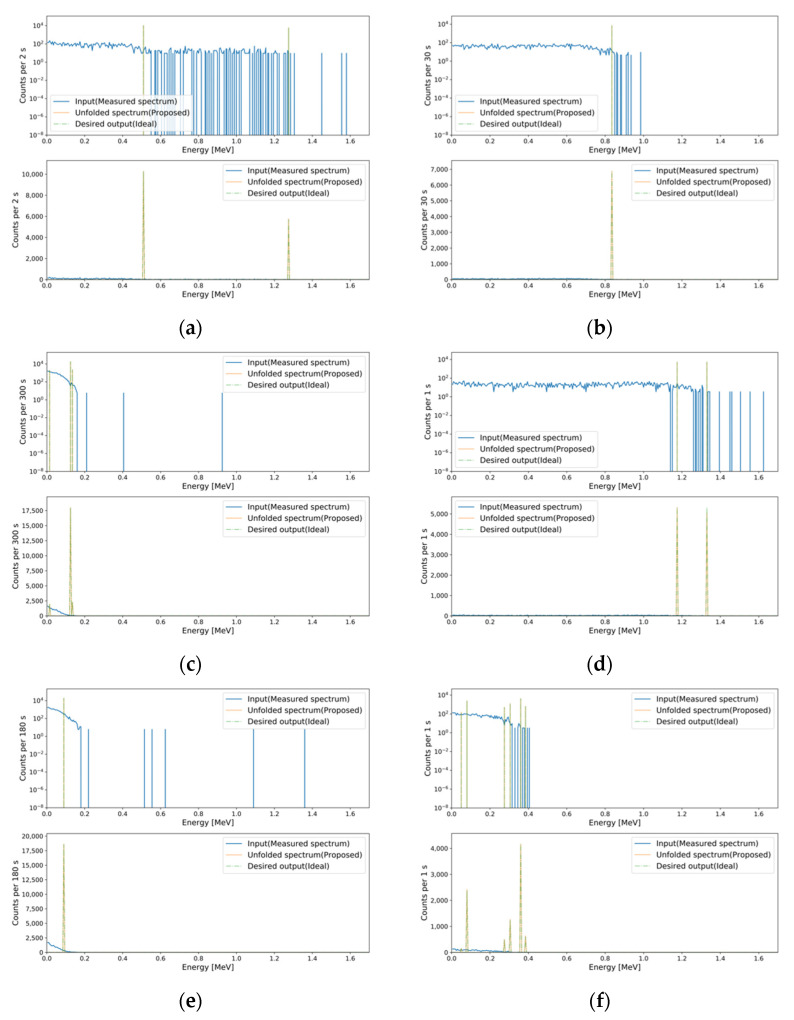

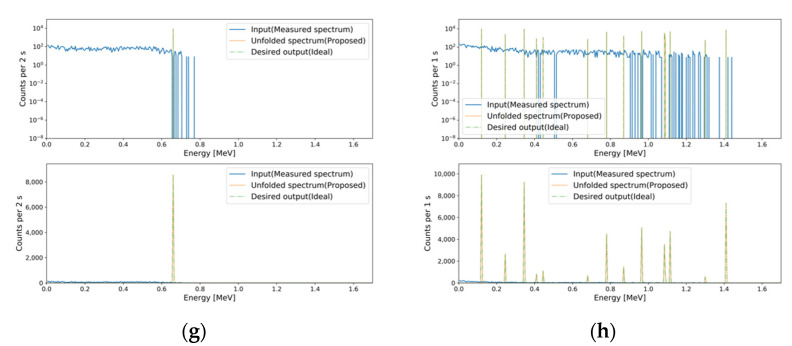
FEP generation results for the measured spectra at each MRC. For the eight gamma−ray sources, the MAPEs are (**a**) 3.999 × 10^−1^ for ^22^Na; (**b**) 1.999 × 10^−1^ for ^54^Mn; (**c**) 5.999 × 10^−1^ for ^57^Co; (**d**) 3.999 × 10^−1^ for ^60^Co; (**e**) 1.999 × 10^−1^ for ^109^Cd; (**f**) 1.999 × 10^−1^ for ^133^Ba; (**g**) 1.999 × 10^−1^ for ^137^Cs; and (**h**) 2.799 × 10^−1^ for ^152^Eu.

**Figure 10:**
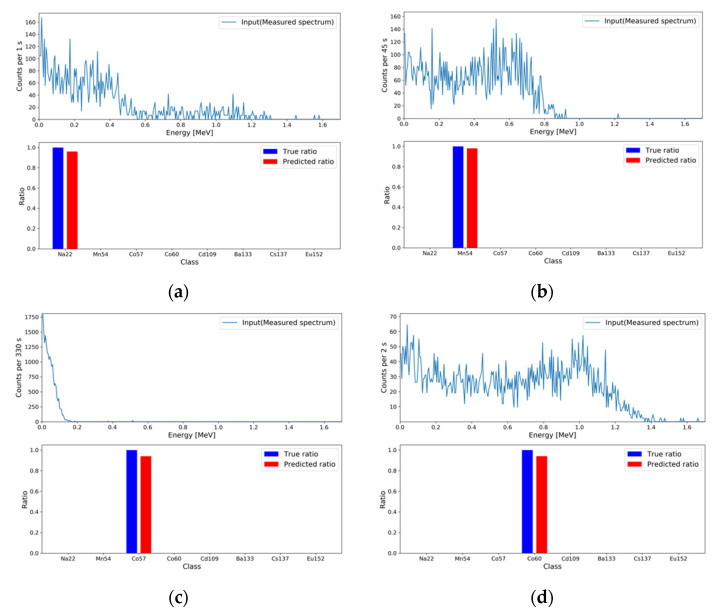

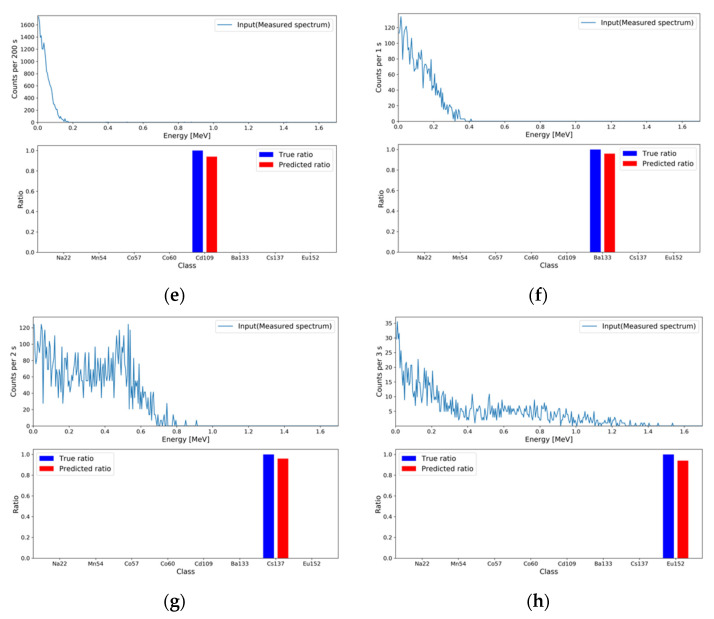
Relative activity regression results for the measured spectra at each MRC. For the eight gamma-ray sources, the MAPEs are (**a**) 5.999 × 10^−1^; (**b**) 2.499 × 10^−1^; (**c**) 7.499 × 10^−1^; (**d**) 7.499 × 10^−1^; (**e**) 7.499 × 10^−1^; (**f**) 5.001 × 10^−1^; (**g**) 5.001 × 10^−1^; and (**h**) 7.499 × 10^−1^.

Figure 9 and Figure 10 show the FEP generation and relative activity regression results, respectively, for the measured spectra with different measurement periods corresponding to each MRC.

### 3.3. Performance Comparison

**Figure 11:**
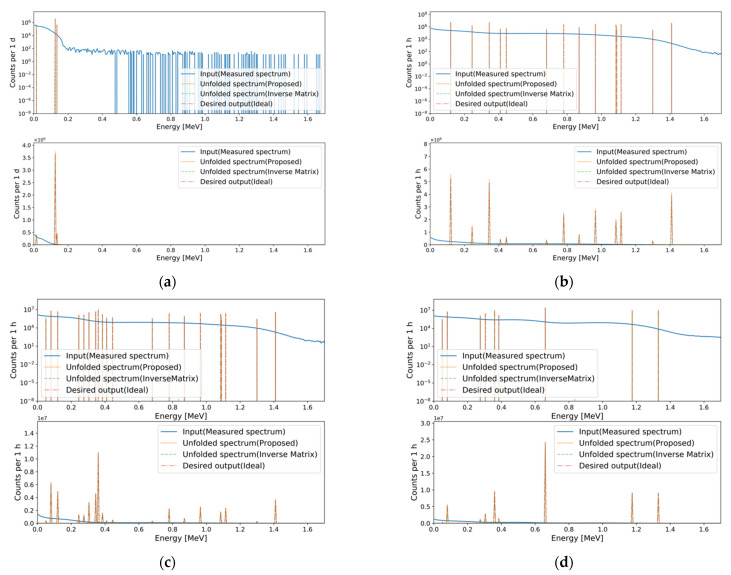
Examples of the unfolding results using the inverse of the transfer matrix and the final MTL model from fine measured spectra, from single to multiple gamma-ray sources. Results of (**a**) ^57^Co; (**b**) ^152^Eu; (**c**) ^133^Ba and ^152^Eu; (**d**) ^60^Co, ^133^Ba and ^137^Cs. The MAPEs of the inverse of the transfer matrix and final MTL model, respectively, are (**a**) 1.428 × 10^−6^ and 1.538 × 10^−2^; (**b**) 3.044 × 10^−5^ and 1.486 × 10^−1^; (**c**) 3.277 × 10^−5^ and 5.752 × 10^−2^; (**d**) 2.590 × 10^−5^ and 6.046 × 10^−2^.

In pseudo-gamma spectroscopy studies, the inverse of the transfer matrix [8] is used to unfold FEPs, and thus identify and quantify the gamma-ray sources in plastic gamma spectra. To compare the unfolding performances of the developed MTL model with that of the inverse of the transfer matrix, we performed FEP unfolding using both methods. Figure 11 depicts several unfolding results using the inverse of the transfer matrix and the final MTL model for fine measured spectra from single to multiple gamma-ray sources. As shown in the figure, both models successfully unfolded the FEPs from plastic gamma spectra. The inverse of the transfer matrix unfolded FEPs with lower MAPE, indicating that the inverse of the transfer matrix unfolds FEPs more accurately.

**Figure 12:**
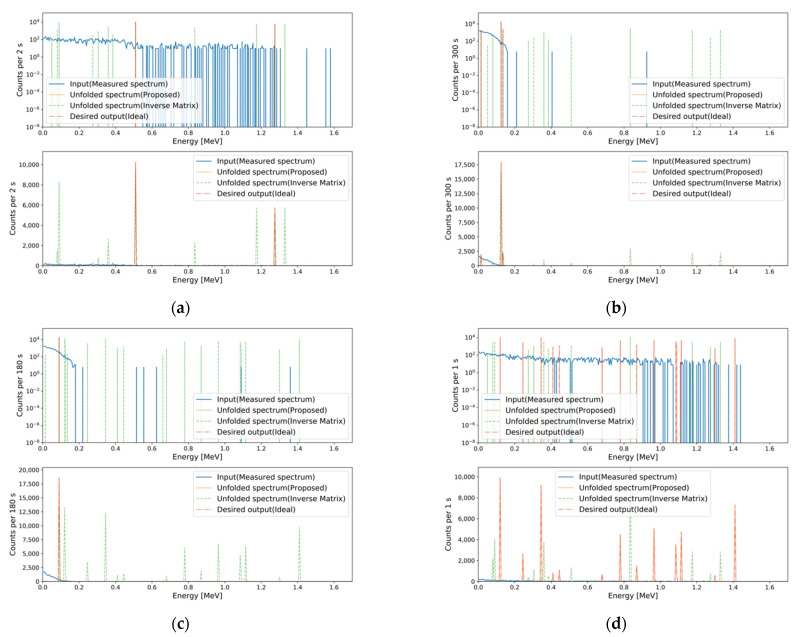
Unfolding results using the inverse of the transfer matrix and the final MTL model for measured spectra whose total net counts are the evaluated MRCs for the generation task. Results of (**a**) ^22^Na; (**b**) ^57^Co; (**c**) ^109^Cd; (**d**) ^152^Eu. The MAPEs of the inverse of the transfer matrix and the final MTL model, respectively, are (**a**) 1.152 × 10^3^ and 3.999 × 10^−1^; (**b**) 2.492 × 10^2^ and 5.999 × 10^−1^; (**c**) 1.483 × 10^3^ and 1.999 × 10^−1^; (**d**) 7.709 × 10^2^ and 2.799 × 10^−1^.

Although the inverse of the transfer matrix unfolds FEPs more accurately than the final MTL model, it cannot be applied to spectra with counting fluctuations. To confirm this, we performed FEP unfolding for the measured spectra whose total net counts (background-subtracted counts) were the evaluated MRCs for the generation task, using both methods. Figure 12 shows several unfolding results for measured spectra having counting fluctuations. Although the MRCs of the gamma-ray sources in Figure 12 were relatively high, the inverse of the transfer matrix failed to unfold the FEPs from measured gamma spectra with counting fluctuations while the final MTL model successfully unfolded the FEPs. Therefore, it was verified that the developed MTL model overcomes this limitation of the inverse of the transfer matrix method, i.e., inapplicability to spectra having counting fluctuations.

## 4. Discussion and Conclusions

In this study, we introduced a deep learning model with MTL for unfolding FEPs and predicting the relative activities of the gamma-ray sources in plastic gamma spectra. To train the deep learning model, we prepared a dataset containing training, validation, and testing sets paired with GEB and FEP spectra. The GEB spectra were generated through MCNP simulation, spectral synthesis, and the random sampling method, whereas the FEP spectra were generated using the gamma energies and their isotope emission intensities. We optimized the hyperparameters of the deep learning model using the Bayesian optimization method. In addition, we proposed a deep learning module. The performance of the final model was verified using the simulated and measured plastic gamma spectra. The results showed that our model successfully unfolded the FEPs and predicted the relative activities of the gamma-ray sources in the simulated and measured plastic gamma spectra.

Moreover, we evaluated the MRCs of each gamma-ray source for both tasks using a dataset generated in the same manner as the dataset created for training the MTL model using the MAPE metric with a criterion of 1%. To confirm the effectiveness of the proposed deep learning module, the MRCs were evaluated and it was confirmed that the final MTL model had generally lower MRCs for both tasks compared to the baseline model. The evaluated MRCs for the final model were 10,270 for ^22^Na, 6870 for ^54^Mn, 17,990 for ^57^Co, 5310 for ^60^Co, 18,590 for ^109^Cd, 3960 for ^133^Ba, 8970 for ^137^Cs, and 15,080 for ^152^Eu. The MRCs for ^57^Co and ^109^Cd were relatively high, possibly because of to the low-energy gamma-ray emission (below 100 keV) of these gamma-ray sources and because ^57^Co and ^109^Cd have highly similar spectra. Owing to the poor energy resolution of the plastic gamma spectra, these low-energy gamma rays are difficult to distinguish.

While it was demonstrated that the proposed MTL model successfully unfolded FEPs and predicted the relative activities of gamma-ray sources from plastic gamma spectra, we are aware of the following limitations in our model: (i) As the machine learning approach is a data-driven method, its effectiveness depends on the dataset. Our model was trained using the generated plastic spectra for eight gamma-ray sources. For the spectrum of an untrained gamma source, our model generated incorrect FEPs and predicted incorrect relative activities corresponding to one or a combination of the trained gamma-ray sources. (ii) The MRCs may vary with the addition of gamma-ray sources. As mentioned above, the MRCs for ^57^Co and ^109^Cd were significantly higher than those of the other sources, possibly due to their highly similar spectra. Hence, if gamma-ray sources with highly similar spectra are included in the dataset, the MRCs of all such sources may increase because more counts are necessary to differentiate between them. (iii) The model performance may depend on the experimental environment. This study addressed the unfolding of FEPs and the prediction of the relative activities of gamma-ray sources from plastic gamma spectra in specific cases where the spectra used for the dataset were simulated and measured in a strictly controlled environment in which there were no temperature variations; the experimental setup was fixed and bare sources were used. In practical situations, the spectra may change because of the surrounding materials in the environment. Furthermore, temperature variations may cause gain shifts in the measured spectra. As indicated in the MRC evaluation results, our model requires a greater number of counts to distinguish similar spectra. Therefore, such gain shifts may decrease the performance of our model. The development of gain shift correction methods [41] could assist in mitigating this shortcoming. Further investigation is required to address these limitations before practical application in radiation portal monitors, radiation monitoring systems, and radiation dosimeters is realizable.

## Figures and Tables

**Table 1:** Details of the used gamma-ray sources.

Source	Activity (kBq)	Reference Date *	Estimated Activity ** (kBq)	Gamma Energy (MeV)	Emission Intensity (%)
^22^Na	385.5	1 June 2017	169.48	0.511	180.76
				1.275	99.94
^54^Mn	341.3	1 June 2017	28.04	0.835	99.98
^57^Co	395.2	1 June 2017	22.37	0.014	9.16
				0.122	85.6
				0.137	10.68
^60^Co	380	1 June 2017	263.42	1.173	99.9
				1.333	99.98
^109^Cd	346.1	1 June 2017	64.04	0.088	3.64
^133^Ba	370	1 June 2017	301.89	0.081	32.9
				0.276	7.16
				0.303	18.34
				0.356	62.05
				0.384	8.94
^137^Cs	378.1	1 June 2017	352.15	0.662	85.1
^152^Eu	385.2	1 June 2017	328.92	0.122	0.87
				0.245	0.96
				0.344	1.09
				0.411	1.09
				0.444	1.11
				0.678	1.29
				0.779	1.41
				0.867	4.25
				0.964	14.6
				1.086	10.21
				1.09	1.73
				1.112	13.64
				1.299	1.62
				1.408	21

* Reference date is the date when the radioactivity was measured by the manufacturer. ** Estimated activity is the calculated radioactivity from the date of measurement. The estimated activities were calculated using Rad pro [32].

**Table 2:** Hyperparameter tuning results.

Parameter	Type	Range	Final Value
Depth of the encoder and decoder layers	Continuous	2–8	6
Decreasing and increasing rates for the # of neurons	Continuous	0.5–0.98	0.836
Regressor layers depth	Continuous	1–4	2
# of neurons in the regressor layers	Continuous	10–300	180
Activation functions in the hidden layers	Discrete	Relu, Sigmoid	Relu
Activation function in the last decoder layer	Discrete	Linear, Sigmoid, Tanh, Exponential	Exponential

**Table 3:** Evaluation results using the test set for the baseline model with deep learning modules. Deep neural network (DNN) with the proposed module was selected as the final multitask learning (MTL) model.

Model	Generation Loss (%)	Regression Loss (%)
DNN (Baseline)	310.678	34.225
DNN + Attention	67.987	34.201
DNN + Attention + Skip	58.704	34.797
DNN + Multi-head self-attention + Skip	124.351	34.397
DNN + Proposed + Skip -> Final model	37.597	34.146
CNN	251.524	34.003
CNN + Attention	82.609	34.148
CNN + Attention + Skip	68.262	34.651
CNN + Multi-head self-attention + Skip	109.087	38.017
CNN + Proposed + Skip	80.216	34.973
DNN (two models)	881.641	34.285
DNN + Proposed + Skip (two models)	309.856	34.148

**Table 4:** Evaluated minimum required counts (MRCs) of both models for the generation and regression tasks.

Source	MRC for Generation		MRC for Regression	
	Baseline MTL	Final MTL	Baseline MTL	Final MTL
^22^Na	7820 ± 89	10,270 ± 102	9070 ± 96	7670 ± 88
^54^Mn	6410 ± 80	6870 ± 83	11,130 ± 105	11,130 ± 106
^57^Co	18,640 ± 137	17,990 ± 135	22,510 ± 150	19,540 ± 140
^60^Co	14,980 ± 23	5310 ± 73	15,130 ± 123	7420 ± 87
^109^Cd	19,990 ± 142	18,590 ± 137	26,710 ± 164	19,240 ± 139
^133^Ba	12,430 ± 112	3960 ± 63	12,430 ± 112	3960 ± 63
^137^Cs	14,330 ± 120	8970 ± 95	14,330 ± 120	8970 ± 95
^152^Eu	16,940 ± 131	15,080 ± 123	16,940 ± 131	15,080 ± 123

## Data Availability

Data of this research is available upon request via corresponding author.
